# Shedding light on biological sex differences and microbiota–gut–brain axis: a comprehensive review of its roles in neuropsychiatric disorders

**DOI:** 10.1186/s13293-022-00422-6

**Published:** 2022-03-25

**Authors:** Parnian Shobeiri, Amirali Kalantari, Antônio L. Teixeira, Nima Rezaei

**Affiliations:** 1 School of Medicine, Tehran University of Medical Sciences (TUMS), Children’s Medical Center Hospital, Dr. Qarib St., Keshavarz Blvd, 14194 Tehran, Iran; 2 Network of Immunity in Infection, Malignancy and Autoimmunity (NIIMA), Universal Scientific Education and Research Network (USERN), Tehran, Iran; 3 Non-Communicable Diseases Research Center, Endocrinology and Metabolism Population Sciences Institute, Tehran University of Medical Sciences, Tehran, Iran; 4 Research Center for Immunodeficiencies, Pediatrics Center of Excellence, Children’s Medical Center, Tehran University of Medical Sciences, Dr. Gharib St, Keshavarz Blvd, Tehran, Iran; 5 Neuropsychiatry Program, Department of Psychiatry and Behavioral Sciences, McGovern Medical School, The University of Texas Health Science Center at Houston, Houston, TX USA; 6 Department of Immunology, School of Medicine, Tehran University of Medical Sciences, Tehran, Iran

**Keywords:** Microbiota–gut–brain axis, Microbiome, Sex difference, Mental disorders, Neurological disorders, Neurodegenerative disorders, Probiotics

## Abstract

Women and men are suggested to have differences in vulnerability to neuropsychiatric disorders, including major depressive disorder (MDD), generalized anxiety disorder (GAD), schizophrenia, eating disorders, including anorexia nervosa, and bulimia nervosa, neurodevelopmental disorders, such as autism spectrum disorder (ASD), and neurodegenerative disorders including Alzheimer’s disease, Parkinson’s disease. Genetic factors and sex hormones are apparently the main mediators of these differences. Recent evidence uncovers that reciprocal interactions between sex-related features (e.g., sex hormones and sex differences in the brain) and gut microbiota could play a role in the development of neuropsychiatric disorders via influencing the gut–brain axis. It is increasingly evident that sex–microbiota–brain interactions take part in the occurrence of neurologic and psychiatric disorders. Accordingly, integrating the existing evidence might help to enlighten the fundamental roles of these interactions in the pathogenesis of neuropsychiatric disorders. In addition, an increased understanding of the biological sex differences on the microbiota–brain may lead to advances in the treatment of neuropsychiatric disorders and increase the potential for precision medicine. This review discusses the effects of sex differences on the brain and gut microbiota and the putative underlying mechanisms of action. Additionally, we discuss the consequences of interactions between sex differences and gut microbiota on the emergence of particular neuropsychiatric disorders.

## Highlights

- The human microbiome is a unique set of organisms affecting health via the gut–brain axis.
- Neuropsychiatric disorders, eating disorders, neurodevelopmental disorders, and neurodegenerative disorders are regulated by the microbiota–gut–brain axis in a sex-specific manner.
- Understanding the role of the microbiota–gut–brain axis and its sex differences in various diseases can lead to better therapeutic methods.

## Background

Sex is defined based on biological attributes and features (e.g., chromosomes, gene expression, hormone levels and function, and reproductive/sexual anatomy) of both human and animal species [1, 2]. Sex differences are prominently reflected in the brain and behavior from birth to adulthood [3]. Sex differences are indicated to be among the main contributors to differences in the frequency of neuropsychiatric disorder, brain areas' structures, and their functionality due to the modifications of sex hormones in males and females [3]. The differences in the incidence of mental illnesses such as major depression and generalized anxiety in men and women may enhance the probability of the significant effects of sex differences in their prevalence. Moreover, sex-specific genes and hormones are slightly suggested to strengthen the impact of socioeconomic status related to sex and gender in the development of neuropsychiatric diseases [3, 4]. Interestingly, the role of sex-differentiated genetic features in the prevalence of neuropsychiatric and behavioral traits was investigated [5, 6]. Martin et al. [6] demonstrated that genetic features could not support the existing sex differences in the presentation of neuropsychiatric disorders. Performing the largest genome-wide genotype-by-sex (G × S) interaction, Blokland et al. [5] found considerable overlap in the genetic factors of the two sexes; however, regarding schizophrenia, bipolar disorder, and major depressive disorder, significant variant- and gene-specific sex differences were observed. Yet, recent research and evidence imply that additional pathophysiological pathways (e.g., sex–microbiota–gut–brain axis) are likely to contribute and explain the existing differences between men and women in the occurrence of brain- and behavior-related disorders [7].

For many years, the dominant models for neuropsychiatric disorders were entirely focused on the nervous system [8], and neuro-explanations of mental health have conquered the last decades of mental health research [9]. Traditionally, microorganisms were not considered critical to the development and function of the CNS. Nevertheless, in recent years, the discovery of the size and complexity of the human microbiome and investigations into the microbiome–gut–brain axis have shaped a critical paradigm shift in neuroscience and mental health [10–12]. Furthermore, sex is a critical factor in several mental health disorders, so investigating the microbiome–gut–brain axis is better to be sex-specific.

Over the last few decades, scientists and researchers have investigated the possible effects of human body microorganisms (especially the gut microbiota) not only on human digestion or gastrointestinal mechanisms but also on the brain and behavior. Gut and brain affect each other in a bidirectional manner via immune, endocrine, and neural pathways [13]. Experimental and clinical studies have shown potential links between gut and CNS, and vice versa, leading to neuropsychiatric disorders [13]. Within this framework of the brain–gut–microbiota axis, the differential development of various neurologic and psychiatric disorders in men and women has been suggested [7]. Evidence indicates that sex has significantly impacted the human microbiome, and subsequently, ‘microgenderome’ was introduced, referring to the interactions between microbiota, sex hormones, and the immune system [14–16]. Notably, sex-specific features are encountered to alter human microbiota patterns [17]. Sex-specific alterations in gut microbiome ecological structure may indicate an adaptation to preserve physiological and behavior differences between men and women throughout life because of their differing nutrient and energy requirements for growth, development, and reproduction [18–21]. Regarding the sex hormone and microbiota interaction, postmenopausal women who consume soy isoflavones (which has structurally estrogen-like metabolites) are more likely to have a Bifidobacterium-enriched gut microbiome, whereas *Clostridiaceae*, a previously unknown genus of bacteria, is reduced in abundance [22]. Interestingly, bilateral ovariectomy has been linked to an increased prevalence of *Clostridium bolteae* in humans [23]. Adults' fat distribution and obesity may also contribute to the observed sex-dependent microbiome variations. Adult men and women, however, show a correlation between fat distribution and *Holdemanella* and *Gemmiger* (phylum Firmicutes), although in the other pattern [24]. In women, android fat ratio was negatively connected with Holdemanella and positively correlated with Gemmiger, but in males, android fat ratio was favorably correlated with Holdemanella and negatively correlated with Gemmiger [24]. In addition, various alterations in the patterns of gut microbiota composition were revealed due to age-associated changes in the genes, and the geographical status [25].

This narrative review summarizes the current literature on how sex differences may affect the gut–brain axis and the underlying pathways. Then, we discussed psychiatric and mental disorders and the potential effects of sex differences in the gut–brain axis, which may play an essential role in the pathogenesis of the following conditions based on clinical and experimental studies: neurodevelopmental disorders, mood and stress disorders, and stress-related functional gastrointestinal disorders (FGIDs). Furthermore, we elaborated on the possible interactions of sex and microbiota–gut–brain axis on the development of neurodegenerative diseases. At last, potential therapeutics based on microbiota-targeted and sex-based dietary interventions are described.

## The gut–brain axis (GBA)

**Fig. 1:**
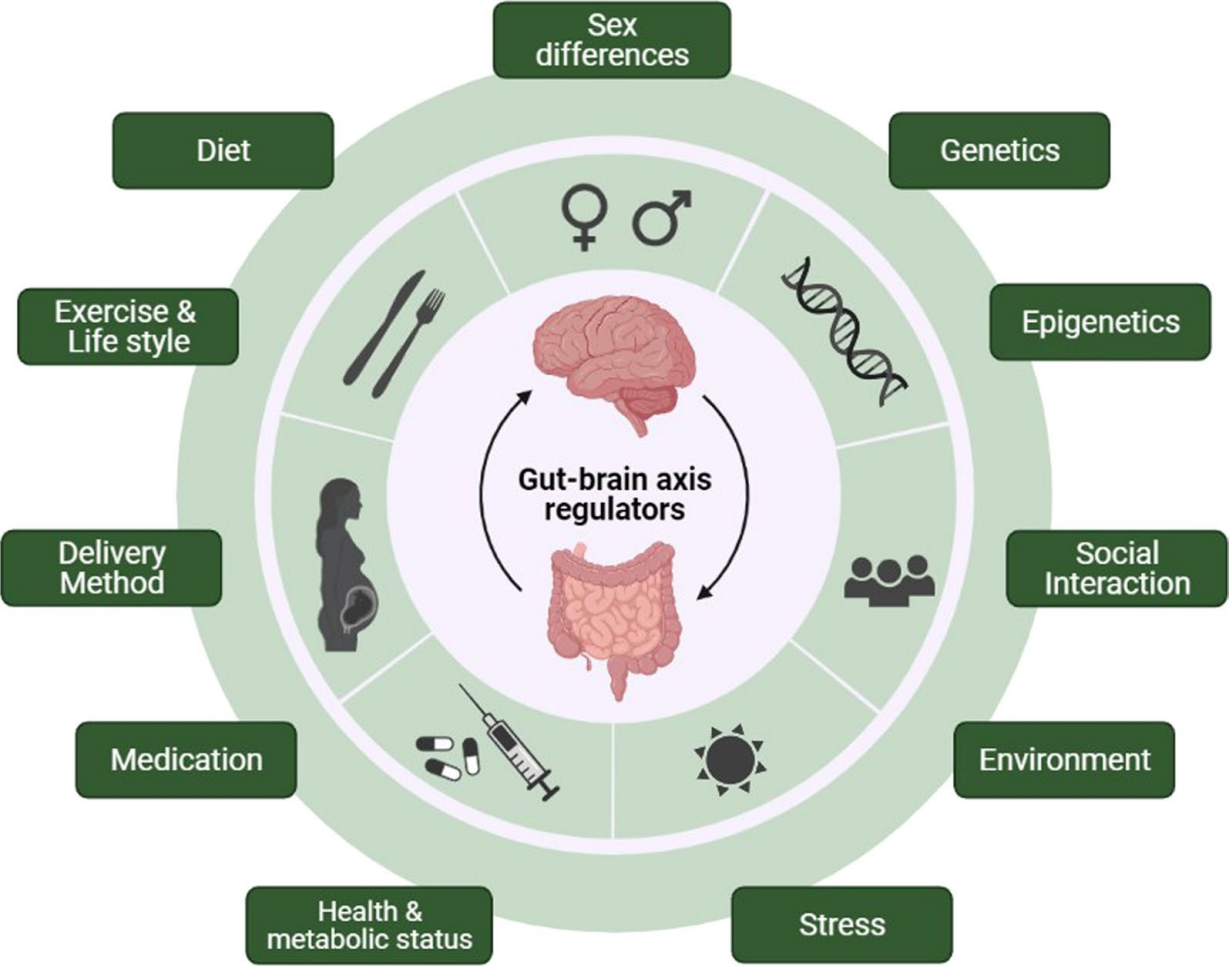
Regulators of gut–brain axis

The gut–brain axis is defined as complex bidirectional communications between various components of the body, including the brain, the sympathetic and parasympathetic divisions of the autonomic nervous system, the endocrine and immune systems, the enteric nervous system, and the gut microbiome [26]. The microbiome refers to the whole genetic material of microorganisms [27]. Several studies reported on the potential mechanisms of microbiome–CNS crosstalk, including endocrine [28], the vagus nerve [29, 30], immune [31, 32] and neuropeptide/neurotransmitter systems [33, 34], and signaling molecules. Considering signaling molecules, they mainly include short-chain fatty acids (SCFA) [35], branched-chain amino acids [36], bile moieties [37], and peptidoglycans [38]. Many factors regulate the gut–brain axis, including sex differences, genetics, diet, etc. We summarize the gut–brain axis regulators in Fig. 1.

### Sex differences in the gut–brain axis

#### Sex differences in the brain

Sexual dimorphism exists in the brain of many vertebrate species, including primate species and humans [39]. Different studies on this matter showed larger total brain volume, more cortical surface area and gyrification, a greater ratio of white matter to gray matter, less gray matter density, and less cortical thickness in males compared to females, as well as other differences in white matter organization, cerebral blood flow, caudate nucleus, and hippocampus size [40, 41]. Different gene regulation and gene expression could be an underlying cause of this dimorphism [39, 42, 43]. There is an active discussion about the magnitude and effect size of sex differences in brain structure and function. For example, studies have controversy concerning the magnitude, location, and direction of sex differences in the human brain's local gray matter volume (GMV) [44]. Trying to find an answer, Lotze et al. [45] applied a Gaussian-process regression coordinate-based meta-analysis including 16 voxel-based morphometry studies in a well-powered sample (n = 2838). They found more GMV in medial and lateral prefrontal areas, the superior temporal sulcus, the posterior insula, and the orbitofrontal cortex in women than men, while more GMV in subcortical temporal structures, such as the amygdala, hippocampus, temporal pole, fusiform gyrus, primary visual cortex, and motor areas (premotor cortex, putamen, anterior cerebellum) in men than women. Ritchie et al. [46] found that males have higher raw volumes, raw surface areas, and white matter fractional anisotropy; females have higher raw cortical thickness and higher white matter tract complexity, with considerable distributional overlap between the sexes.

One attractive mouse model for learning potential mechanisms of sex differences is using the four core genotypes (FCG) model. This model includes mice where sex chromosome complement (XX vs. XY) is unattached to the animal’s gonadal sex. The four genotypes are XX gonadal males or females and XY gonadal males or females. As Arnold et al. stated, this model allows one to measure the differences in phenotypes caused by sex chromosome complement (XX vs. XY), the differential effects of ovarian and testicular secretions, and the interactive effects of these two [47]. Using this model could be a helpful strategy to investigate whether the role of the microbiome and the gut–brain axis on the brain is genotype-dependent or not.

Sex differences in the brain are minor at birth, but quickly reach adult levels. [48]. Interestingly, hippocampal plasticity was found to be affected by sex-related alterations of the microbiome. Darch et al. [49] studied germ-free mice (mice with no microorganisms living in or on them) and found that microbiota absence may lead to changes in dendritic signaling integrations in the CA1 region, which is the first region in the hippocampal circuit, from which a major output pathway goes to the entorhinal cortex [50]. Additionally, CA1 has been shown to have significant roles in autobiographical memory, mental time travel, and autonoetic consciousness in humans [51]. It could be implied that microbiome alterations can affect these roles in humans, but further studies are needed to assess its relationship.

#### Sex differences in microbiome

A complex community of archaeal and bacterial cells, including more than 1000 species, covers the human gastrointestinal tract, called the human gut microbiota [52]. Bacteroidetes, Firmicutes, Actinobacteria, Verrucomicrobiota, Fusobacteria, and Proteobacteria are the predominant bacterial phyla. The most common species are *Bacteroides fragilis*, *Bacteroides melaninogenicus*, *Bacteroides oralis*, *Enterococcus faecalis*, and *Escherichia coli*. *Methanobrevibacter smithii* and *Methanosphaera stadtmanae* are the dominant archaeal species. Other microbes are present, such as protozoans, fungi, bacterias, and viruses [53, 54].

Animal studies on mice show significant differences in gut microbiota composition according to sex [55, 56]. *Lactobacillus plantarum* and *Bacteroides distasonis* are more common in B6 female mice than B6 males, while Bifidobacterium is more common in BALB/c female mice compared to BALB/c males [55]. Human studies show that each individual has a unique microbiome composition with a core microbiota shared in everyone [52, 57, 58]. Different factors such as sex, age, and body mass index (BMI) can affect this composition. Studies show that male and female microbiota differs in bacterial phyla level, at the genus level, and the species level, with greater diversity in females [59–61].

#### Sex-by-diet interactions in microbiome

Diet and the composition of the gastrointestinal microbiome affect each other in children and adults [53, 62–64]. For example, having an animal-based diet increases abundance of bile-tolerant microorganisms (Alistipes, Bilophila, and Bacteroides) and decreases levels of polysaccharides metabolizers (Roseburia, *Eubacterium rectale*, and *Ruminococcus bromii*) [53]. Conversely, intestinal bacteria can control preferences, appetite, and feelings of satiety [63, 65, 66]. Sex differences also have an impact on metabolism. For instance, the presence of XX chromosome complement in a cell will result in not expressing the 78 protein-coding genes, nor an unknown number of noncoding RNAs present on the Y chromosome [67]. Chen et al. [68] demonstrated that both gonadal sex and sex chromosome complement had an influence on body weight in gonadally intact mice of the four core genotypes. Adults were then stripped of their gonadal glands, thereby eradicating the acute effects of gonadal hormones. Regardless of their initial gonadal sex, XX mice, given a conventional mouse chow diet (~ 5% fat), had almost twice the fat mass of XY mice. After gonadectomy, the difference between XX and XY mice was accentuated, with XX mice gaining weight more quickly and diverging from XY mice after just three days on the high-fat diet. Specific fat depot analysis indicated that XX mice had bigger subcutaneous inguinal adipose tissue depots than XY mice did. These finding show that the sex chromosomal complementation has a role in the observed disparities in fat distribution between females and males. Other mechanisms also exist, but their details are beyond the scope of its context.

Different murine studies uncovered that the interaction between microbiome and diet is sex-dependent in various conditions [69–72]. For example, high-fat diet-induced body weight gain was higher in male mice than females, and only males developed hepatic steatosis, insulin resistance, glucose intolerance, and thigh muscle loss [69, 70]. Moreover, male mice treated with antibiotics before a high-fat diet developed insulin resistance, whereas female mice had a higher fasting glucose level. Male mice's gut microbiota was significantly different from that of female mice, regardless of their diet. More Lactobacillus, Bifidobacterium and Parabacteroides genera were found in females than in men. Short-chain fatty acid-producing bacteria such as *Roseburia* and *Lachnospiraceae NK4A136* group were less abundant after HFD feeding, which had an effect on the gut microbiota composition. Antibiotics followed by HFD altered the gut microbiota in distinct ways in men and females, showing that antibiotic susceptibility is sex-dependent [69]. In this regard, Bolnick et al. [21] experimented on fish species. They reached several findings: (1) some differences in microbial populations among individuals can be explained by their diet, and (2) dietary impacts are highly dependent on sex. Moreover, men's and women's microbiota have distinct effects on each other regarding food, whether we investigate across microbial PCoA (principal coordinate analysis) axes or individual OTUs (operational taxonomic units). Furthermore, they revealed that microorganisms responded more strongly to the food of stickleback females than to the diet of males. According to their findings, the microbial taxa displaying diet (or sex*diet) impacts in the lab were not the same as the microbial taxa showing diet (or sex*diet) effects in the wild (natural) stickleback [73]. Interestingly, even while nutrition may influence the microbiome in a predictable way, and while certain bacteria found in the intestines of various people can alter particular dietary components in a predictable way, many food-microbe interactions are unpredictable and unique [74, 75].

#### Sex hormones and gut microbiota interactions

Sex differences in gut microbiota significantly appear after puberty [1]. This supports the idea that sex hormones may play a role in the gut microbiota composition [14, 76]. In an experimental study, male and female mice did not show any significant differences in gut microbiota before puberty, while male mice revealed deviations in the gut microbiota components after puberty. Moreover, castration in male mice to remove androgen sources caused gut microbiota composition in castrated male mice to become similar to female ones [76]. Castration also changes metabolism and feeding behavior, in ways that are dependent and independent of androgen. All of these systemic adjustments may also have an impact on the composition of the gut microbiota [77, 78]. In addition, fecal microbiota transplantation (FMT) corroborates the role of sex in the gut microbiota composition. Accordingly, results of an investigation into the Denaturing gradient gel electrophoresis (DGGE) and terminal restriction fragment length polymorphism (T-RFLP) profiles of fecal microbiota from specific pathogen-free (SPF) and human flora associated (HFA) rats indicated that variance over time was less important than variation between individuals, and that phylogenetic profiles aggregated according to the sex of the rats studied [79]. It has been suggested that commensal bacteria affect the metabolism in male species by regulating the production and utilization of testosterone [1].

Notably, gut microbiota and estrogen are believed to have bidirectional interactions [80]. Several reports on bilateral ovariectomy reveal that it may lead to microbial dysbiosis in mice and increased *Clostridium bolteae* [23, 56, 81]. Furthermore, the abundance of fecal Clostridia (e.g., non-Clostridiales and three genera in the Ruminococcaceae family) is positively associated with non-ovarian systemic estrogen levels [80]. Interestingly, gut microbiota regulates estrogen levels by mediating its metabolism in the enterohepatic circulation [80]. Other than sex hormones, diet, drugs, body mass index, and colonic transit time are factors that enhance the influence of sex differences on human gut microbiota [1].

### Common mechanisms of interaction

#### Interactions between the CNS and gut microbiota

Stress affects the composition of the gut microbiota in two directions: emotionally and physiologically. The *Lactobacillus* population was decreased in a mouse model after only two hours of social disturbance [82]. Interestingly, the *Lactobacillus* abundances in the feces of rhesus monkeys dropped after they were separated from their mothers at the age of 6–9 months [83]. In another investigation, *Lactobacillus* concentration in feces was shown to be lower in stressed students than in less stressed individuals [84]. Intestinal microbial reproduction is boosted by prebiotics and dietary fibers, yet stress affects mucus production patterns [85]. In dogs, the gastrointestinal postprandial motility is affected by audio stress, which results in a decline in gastric evacuation [85]. Also, stress-induced alterations in intestinal motility and gut microbiota structure are observed in mice when their mothers are separated from them [86]. Stress mediators increase intestinal permeability, which in turn alters the germ characteristics, causing local immunological activation [87, 88]. Bacteroides and Clostridium species were decreased in the cecum of adult mice exposed to prolonged stress. Additionally, the immune system was stimulated, with increased levels of interleukin-6 and C–C chemokine ligand 2. When acute stress triggered the release of corticotropin-releasing hormone (CRH) in the CNS, mast cells, which have a high affinity for CRH, were activated. This enhanced gastrointestinal and blood–brain permeability [89]. Toxins and lipopolysaccharides (LPS) found in the gut may reach the systemic circulation and the CNS when mast cells are activated by chronic stress.

#### Gut microbiota affecting the CNS

##### Immune regulation

When gut microbiota cause an infection, the infected cells may reach the CNS and trigger inflammatory responses. The immune system is impaired further when cytokines are produced into the bloodstream as a result of chronic low-grade inflammation. Inflammatory chemicals may be found in the gut microbiota. LPS and peptidoglycan, for example, are two common inflammation-inducing agents. The Toll-like receptor-4 (TLR-4), found on a broad variety of brain cells including monocytes, macrophages, and microglia, recognizes LPS. For instance, the gut microbiome has been shown to activate TLR-4-mediated inflammatory responses in IBS patients who are also depressed [90, 91]. Proinflammatory and anti-inflammatory cytokines may be altered by the actions of gut microbes and probiotics on the innate immune system, which has a direct influence on brain processes. For example, macrophages were activated and infiltrated by *E. coli*, which led to an increase in the production of IFN-γ [92].

##### Modulation of afferent nerves

The vagus nerve may be activated by the gut microbiota, and this stimulation has a significant impact on the brain and on the way people behave. The vagus nerve is a vital element of the sensory route that connects the gastrointestinal tract to the brain. The evidence that the gut microbiota impact the vagus nerve is progressively growing. Gram-negative bacteria have an outer membrane that is mostly composed of LPS. A cytokine known as IL-1β is activated by LPS, which in turn causes the vagus nerve to become inflamed. Moreover, vagotomy prevents the production of cytokines [93, 94]. There is a surge in c-Fos expression (an indicator of neural activity of vagal sensory ganglia and solitary neurons) in mice following *C. jejuni* injection, as shown by Goehler et al. [95] In the mentioned study, neural activity rose quickly after being infected with *C. jejuni*, but there was no rise in proinflammatory cytokines at the same time. This shows that bacteria can significantly affect behavior and attitude through the vagus nerve. Earlier research has shown that Campylobacter can cause anxiety-like behavior if it is caught early. This is because vagally mediated neural circuits are affected [96]. Bravo et al. found that the vagus nerve plays a significant role in the bidirectional connection between the brain and the gastrointestinal tract [29]. The probiotic Lactobacillus rhamnosus decreased anxiety and depression in rats and mice, and this reduction was associated by a decrease in gamma aminobutyric acid (GABA) receptor subunit mRNA expression and corticosterone concentrations. However, Lactobacillus rhamnosus therapy had no impact on the rats or mice that had been vagotomized.

##### Tryptophan metabolism

Tryptophan is a precursor amino acid that is required for the synthesis of serotonin and kynurenine. A deficiency of tryptophan has been linked to clinical depression [97]. Tryptophan metabolism seems to be influenced by gut microbiota. In comparison to regularly colonized mice, germ-free animals had higher serum levels of tryptophan and lower levels of serotonin, indicating that tryptophan hydroxylase expression in the intestines may be decreased [98, 99]. Studies on animals have shown that supplementation with the probiotic *Bifidobacterium infantis* increases levels of inflammatory markers as well as tryptophan and decreases the kynurenine to tryptophan ratio [100].

##### Microbial metabolites (short-chain fatty acids)

Short-chain fatty acids (SCFAs) such as acetic acid, propionic acid, and butyric acid are produced by the gut microbiota. These SCFAs act as histone deacetylase inhibitors [101] and bind to G protein-coupled receptors to activate intracellular signaling [102]. SCFAs therefore serve as intermediaries between the brain and the microbiota and contribute to the processes through which gut microorganisms influence brain physiology and behavior. The microbial-derived SCFAs butyric acid and propionic acid stimulate the gene expression of tyrosine hydroxylase, an enzyme that controls the rate of synthesis of dopamine and noradrenaline, and dopamine-β-hydroxylase, an enzyme that converts dopamine to noradrenaline [103]. GABA, serotonin, and dopamine levels were reduced in germ-free rats after long-term therapy with propionic acid [104]. Thus, microbial-derived SCFAs have a direct impact on both physiology and behavior because of their involvement in a neuronal circuit. Glyconeogenesis gene expression was triggered through an inter-intestinal circuit involving the fatty acid receptor FFAR3 in the gut and the brain [105]. Glial cells, such as microglia and astrocytes, may be affected by SCFAs produced by gut microbes, although the exact mechanism by which SCFAs alter CNS function is still unknown. Butyric acid has been shown to have an anti-inflammatory effect on LPS-induced microglial cells in rats [106]. Additionally, microglial malformation and immaturity were restored in SCFA-treated germ-free mice after activation of FFAR2 [107]. de Almeida et al. demonstrated that GFAP (glial fibrillary acidic protein) was elevated in cultured astrocytes in rats treated with propionic acid [108]. Also, cognitive and sensorimotor impairments were observed when rats were injected with bacteria-derived propionic acid [109].

##### Microbial neural substrates

Bacteria are capable of producing a variety of neurotransmitters and related chemicals. Certain types of intestinal microbiota are capable of producing and releasing neurotransmitters such as GABA, serotonin, catecholamine, and histamine on a local level. These neurotransmitters generated from bacteria can communicate with the CNS through enterochromaffin cells and enteric nerve receptors.

GABA, a significant inhibitory neurotransmitter in the CNS linked with depression, anxiety, autism, and schizophrenia, is generated effectively in the human intestines by *Lactobacillus brevis* and *Bifidobacterium dentium* [110]. Takanaga et al. hypothesized in their animal investigation that gut bacteria-produced GABA passes the blood–brain barrier (BBB) and reaches the CNS [111]. *Lactobacillus rhamnosus* has been shown to alleviate anxiety and depression-related behaviors in mice and to boost the hippocampus's GABA levels [29, 112]. Intestinal microbiota may indirectly influence GABA signaling through the vagus nerve, given that such effects are only visible when the vagus nerve is intact.

Microorganisms in the gastrointestinal tract synthesize dopamine and noradrenaline, two neurotransmitters that affect the CNS. Germ-free mice exhibit significantly lower amounts of noradrenaline and dopamine in their cecums vs SPF mice, suggesting that the gut microbiota may provide catecholamine [113]. Moreover, the rate-limiting enzyme for the production of noradrenaline and dopamine, tyrosine hydroxylase, is found in several bacterial species [114]. Dopamine is synthesized in cultured Lactobacillus bacteria [115]. Dopamine formed in the periphery cannot pass the BBB, hence there is no evidence that microbes produce catecholamines that effect the CNS. While tyrosine (the rate-limiting substrate for noradrenaline and dopamine synthesis) levels are lower in germ-free animals as compared to those in ex-germ-free mice, this indicates that the gut microbiota raises dopamine levels in the brains of germ-free mice [116]. Ex-germ-free mice had higher amounts of catecholamines in their brains than germ-free mice, but the gut microbiota was able to adjust these levels via dopamine and noradrenaline metabolism in the brains of these animals [117].

Histamine, a neurotransmitter and immunomodulator, has a role in the control of critical activities such as waking, cognition, circadian rhythm, and neuroendocrine regulation [118]. Certain gut microbiota are capable of producing histamine. *Lactobacillus reuteri* produces histamine through the expression of the histidine decarboxylase gene [119]. Not only does histidine boost histidine decarboxylase expression in *Lactobacillus reuteri* cultures, but it also increases histamine synthesis. Additionally, via generating histamine, *Lactobacillus reuteri* suppresses the proinflammatory cytokine TNF-α in myeloid progenitor cells. Histamine has been shown to have an immunomodulatory effect on intestinal lymphoid organs, where it is involved in the regulation of Yersinia enterocolitica infection [120]. Furthermore, it has been shown that blocking the H2 receptor decreases mucus production and exacerbates intestinal barrier dysfunction, which may lead to microorganisms being transported into the intestinal lumen through the circulatory system [121].

## Psychiatric disorders

### Definition and classification

A mental or psychiatric disorder is described as an illness with psychological or behavioral manifestations that can cause substantial distress and Functional impairment. No objective tests are available for the decisive diagnosis of mental disorders, and we measure these disorders in terms of deviation from some normative concept [122, 123]. It is not always easy to define normality and abnormality, but different criteria have been proposed, primarily relying on signs, symptoms, and other subjective measures [124, 125]. Various ways exist to classify different types of mental disorders, such as the 5th edition of the Diagnostic and Statistical Manual of Mental Disorders codes (DSM-5 codes) maintained by the American Psychiatric Association (APA) [126] or the clinical modification of the 10th revision of the International Classification of Diseases (ICD-10-CM) supported by World Health Organization (WHO) [127]. A mental or psychiatric disorder is described as an illness with psychological and/or behavioral manifestations that can cause substantial distress and functional impairment. No objective tests are available for the diagnosis of mental disorders [122, 123], but different criteria have been used, primarily relying on signs, symptoms, and other subjective measures [124–127].

Mental disorders have a significant burden on health and economic and social consequences in all countries, and approximately 970 million people worldwide were affected by it in 2017, with anxiety disorder being the top condition [128, 129]. In 2019 depressive disorders and anxiety disorders were in the top ten causes of disease burden worldwide. Their related burden affects women more than men [130]. In 2020, the emergence of the COVID-19 pandemic led to exacerbated many determinants of poor mental health. Daily SARS-CoV-2 infection rates and reductions in human mobility were associated with a 27·6% increase in the prevalence of major depressive disorder and a 25·6% increase in the prevalence of anxiety disorders [131].

### Effect of the gut microbiome and sex differences on psychiatric-related conditions

#### Neurodevelopmental disorders and the microbiome

##### Autism spectrum disorders (ASDs)

Autism spectrum disorder (ASD) is a neurodevelopmental disorder that includes autism, Asperger’s syndrome, and other non-specified pervasive developmental disorder [132]. The symptoms include a lack of social interaction and communication skills, limitation of activity and interests, and repetitive behavior [133]. WHO estimated the worldwide prevalence of ASD at around one in 270 people [134], and it is more prevalent in males with a male-to-female ratio close to 3–4:1 [135, 136].

In ASD patients, the gut–blood barrier is more permeable [137]. As a result, bacterial metabolites and neurotoxic xenobiotics (compounds foreign to a living organism) leak into the patient’s body, setting off new responses such as gut inflammation affecting the brain through altering cytokine levels or altering metabolism [138–140]. GI problems are common in autistic individuals, and ASD is also associated with dysbiosis, specifically with a higher abundance of Clostridiales and an increase of Sutterella and Ruminococcus populations [141]. Still, it is not clear whether dysbiosis is a factor causing ASD or if the disease is causing microbial alterations [138]. Recently, Yap et al. [142] investigated an exciting theme: the potential confounding factors related to the gut microbiome and ASD. They stated that the microbiome has a minor direct association, and instead, ASD findings are associated with a less-diverse diet and sequentially reduced microbial diversity. Despite scientists' attempts, no significant sex-specific ASD signature has been identified in humans. However, Wang et al. investigated differences in gut microbiome-associated epitopes in autistic children and found that sex was associated with specific gut microbiome-associated epitopes. They stated that the diversity of the epitopes differed between males with ASD compared with normal controls, but it was the same in females in the two groups [143]. Some animal studies provide evidence telling that the alteration in gut microbiota is sex-related, with Bacteroides, Parabacteroides, Sutterella, Dehalobacterium, and Oscillospira genera being the key drivers of sex-specific gut microbiome profiles [144]. This alteration can cause broad diversity of behavioral deficits that are characteristics of ASD in mouse models [145]. Other studies also indicate that dysbiosis creates a vicious cycle, affecting the immune system by reducing detoxification and adsorbing toxins, xenobiotics, and neurochemical compounds. Some enterotoxins and food-xenobiotics promote male-specific neurotoxicity, which can account for the higher prevalence of the disease in males, along with intrinsic male immune system vulnerability [139]. One new study on pregnant women found that flu in individuals who did not receive an antibiotic during pregnancy significantly increased the odds of ASD in the child. In this subgroup, the male sex was also associated with increased probabilities of ASD [146].

It may be possible to improve treatment outcomes by restoring the balance of the microbiota–gut–brain axis in autism. Some studies show promising results for treating ASD patients with probiotics or fecal microbiota transplant therapies [141, 147, 148]. One clinical trial in 18 ASD children involving fecal microbiota transplant led to significantly improved GI symptoms and ASD-relevant behaviors, which persisted at the 8-week follow-up [149]. Still, more clinical trials are needed to assess these treatment methods' effectiveness [145].

##### Schizophrenia

Schizophrenia is a severe chronic neurodevelopmental disorder characterized by delusions and hallucinations, negative symptoms, and cognitive dysfunction [150, 151]. The global prevalence of schizophrenia is estimated at around 0.3% [128]. As for other major psychiatric disorders, no laboratory tests are available for schizophrenia, and diagnosis is based only on symptoms [127].

Different studies have shown altered intestinal microbiota is in these patients [29, 107, 152, 153], with changed interkingdom interactions between bacteria and fungi [154]. An increased abundance of Proteobacteria and Chaetomium and a decreased abundance of SCFA-producing bacteria (e.g., Faecalibacterium and Lachnospiraceae) and Trichoderma were recorded [154]. Also, the pro-inflammatory and anti-inflammatory cytokine levels are high in patients, and robust induction of M1 cytokines (e.g., GM-CSF and IL-6) and lack of an IL-2 response point that a bacterial agent could be a trigger [155]. Tracking Intestinal microbiota changes in the patients and comparing them with healthy controls may be a helpful way to develop a biomarker for diagnosis and prognosis, with the genus levels of Eisenbergiella, Ruminococcaceae, and Turicibacter being more efficient as biomarkers [156].

Furthermore, intestinal microbiota significantly influences the oral bioavailability and half-life of antipsychotic medication‌, the first-line treatment for schizophrenia. This reduced bioavailability may explain, at least in part, the poor treatment effect in 30–40% of patients [157]. As a result, switching routes of administration (e.g., parenteral antipsychotics) rather than switching drugs may be a better solution for increasing treatment effectiveness [157, 158]. Altering the microbiome may be an alternative way, but we need clinical trials to evaluate its effectiveness [159].

##### Attention deficit hyperactivity disorder (ADHD)

ADHD is a heterogeneous neurodevelopmental disorder with a childhood-onset that persists into adulthood [126]. A systematic review and meta-regression analysis by Polanczyk et al. and another meta-analysis by Simon et al. estimated the global prevalence of ADHD at around 5% in children and 2.5% in adults, with a female to male ratio of 2–3 in children and approximately 1 in adults. [160–163]. Sex differences are apparent in the prevalence of ADHD and types of its comorbid disorders (such as ASD, tics, learning disorders, rule-breaking behaviors, substance use disorders, mood and anxiety disorders, bipolar disorder, and emotional liability) [160, 164, 165].

It has been theorized that dietary patterns can be linked to ADHD susceptibility via changes in the gut microbiome community. One study revealed that the ADHD group manifested a more significant proportion of refined grains intake and a lower proportion of dairy and vitamin B2. This study also pointed that the relative abundance of Bacteroides coprocola is higher in ADHD patients, while the relative abundance of Bacteroides uniformis, Bacteroides ovatus, and *Sutterella stercoricanis* are lower compared to healthy controls. *S. stercoricanis* showed a significant connection with dairy intake, nuts/seeds/legumes, ferritin, and magnesium. [166].

#### Mood and stress disorders and the microbiome

##### Generalized anxiety disorder (GAD)

Generalized anxiety disorder (GAD) is persistent, inappropriate, and excessive worrying, not limited to specific occasions [167]. GAD is the most common type of anxiety disorder and has a lifetime global prevalence rate of 3.7% [168]. The prevalence of GAD is approximately twice in women than men, and it is concluded that the condition has a sex-specific characteristic [169].

Different studies demonstrated that the gut microbiome is changed in this disorders, and it has been hypothesized that the gut–brain axis could have a role in the pathogenesis of the disease [4, 170–173]. Geary et al. recently investigated whether gut dysbiosis' effects on anxiety-related behaviors are sex-specific via oral administration of a moderate dose of nonabsorbable antimicrobial medications (ATMs: neomycin, bacitracin, and pimaricin) on C57BL/6N mice, and by comparing the results with controls, they concluded that dysbiosis is seen in both sexes, with more substantial effects in females. They also found sex-specific effects on behavior and neuroanatomy, with males being more susceptible than females to microbial modulation of locomotor activity and anxiety-like behavior and females being more susceptible than males to impairments in aversive learning and cued recall [174].

The abundance of Firmicutes and Tenericutes phyla are lower in GAD patients, and the abundance of *Eubacterium coprostanoligenes*, Ruminococcaceae, and Prevotella negatively correlate with anxiety severity were as the abundances of Bacteroides, Escherichia, and Shigella correlate positively with anxiety severity [170]. Regarding the microbiome changes in remission of GAD patients, some contradictory evidence exists: Chen et al. found that altered gut-microbiome profile contributes to the pathogenesis and remission of GAD. At the same time, they remarked that they could not establish a causal association between microbial changes and disease remission due to the small sample size [170]. Navarro‐Tapia et al. proposed that remission did not affect the relative abundance of the various taxa [172].

##### Major depressive disorder (MDD)

Major depressive disorder (MDD) is characterized by a depressed mood, loss of interest or pleasure for at least two weeks [126]. A systematic review by Gutiérrez-Rojas et al. estimated the lifetime prevalence of MDD ranging from 2 to 21%, with a higher prevalence in European countries and lower prevalence in Asian countries [175]. The global prevalence of MDD was reported at around 2% in 2017 [176].

MDD, like GAD, is also twice as prevalent in women than men [177], which reveals a sex-specific characteristic for both conditions. Earlier studies have justified this difference by the role of gonadal steroid hormones, and sex-linked genes have a role in shaping sexually dimorphic brain features [178, 179].

Microbiota changes in MDD patients compared with healthy controls include significant reductions in several taxa at the family and genus levels, specifically in families Prevotellaceae, genus Corprococcus, and Faecalibacterium [180].

One study specifically looked into the sex-specific changes of gut microbiota in individuals with MDD. It showed that female patients had a higher abundance of Actinobacteria and males had a lower abundance of Bacteroidia than sex-matched healthy volunteers [181]. Another study indicated that exposure to probiotics during puberty reduces stress-induced vulnerabilities to emotional behaviors later in life, in a sex-specific manner [182]. Also, it is worth noting that there is a high co-occurrence rate between anxiety, depression, and IBS; but the mechanism causing this comorbidity remains unclear [183, 184].

##### Post-traumatic stress disorder (PTSD)

Post-traumatic stress disorder (PTSD) is characterized by trauma-related and stress-related symptoms in four diagnostic clusters (intrusion, avoidance, negative alterations in cognitions and mood, and alterations in arousal and reactivity), which can impair psychosocial functioning notably [126]. The lifetime prevalence of PTSD ranges from 6.1% to 9.2% in the United States and Canada and around 2% in middle-income countries [185–188]. The reason for lower prevalence outside North America is not fully understood [189], but it may involve environmental risk exposure and cultural issues [190].

An animal study on BALB/c mice has shown that particular strains of Bifidobacteria (e.g., Bifidobacterium longum) can enhance cognitive processes and affect fear learning [191]. The phyla Firmicutes and Bacteroidetes are vulnerable to PTSD-eliciting stress, and the Firmicutes/Bacteroidetes ratio increases due to stress induction in rodents [192]. One new study investigating early-life stress in rats subjected to maternal separation also confirmed gut microbiota alterations. In both sexes, the relative abundance of the Bacteroides genus was increased, and the abundance of the Lachnospiraceae family was decreased. In contrast, maternal separation increased that of Streptococcus genus and decreased Staphylococcus genus only in males; simultaneously, the abundance of the Sporobacter genus was enhanced, and Mucispirillum genus was reduced only in females [193].

Limited human studies have looked into the role of the gut microbiome in PTSD, and the findings are conflicting [194–196]. One study on cirrhotic veterans with PTSD revealed a reduced microbial diversity (with a statistically significant difference compared with the control group), increased relative abundance of Enterococcus and Proteobacteria, and reduced Ruminococcaceae and Lachnospiraceae [197].

#### Eating disorders

##### Anorexia nervosa (AN)

Anorexia nervosa is characterized by insufficient food intake and poor diet, which leads to very low body weight with an indifference to the seriousness of the illness [126]. Anorexia nervosa is more widespread in females, with the prevalence rates estimated at around 0.3–1% in women and around 0.1–0.3% in men in developed countries [198, 199]. Anorexia nervosa (along with bulimia nervosa) has the highest mortality rate of any psychiatric disorder and highest suicide risk of any eating disorders [200, 201]. In addition, comorbidities with autism spectrum disorders have been reported [202].

Besides external environmental factors, genetics and intestinal microbiome may play essential roles in the emergence and progression of the disease [203]. Some researchers investigated the role of the gut–brain axis in anorexia nervosa [204–208]. One study specifically revealed high plasma levels of bacterial ClpB (caseinolytic proteinase B) in the patients [209]. The gut microbiome in these patients differs from healthy individuals because of variations in patients’ intestinal environment (e.g., chronic caloric restriction, macronutrient imbalance, micronutrient deficiencies, fluctuating food availability, osmotic perturbation, and high fiber content) [210–214]. Most, but not all, microbiome studies in AN patients stated dysbioses, such as increased *Clostridium*, *Enterobacteriaceae*, and *M. smithii* species and decreased *Roseburia* species [215–219]. Some studies also have reported a decreased alpha diversity in AN patients [205, 206, 220], while others have not [216, 217, 221].

##### Bulimia nervosa (BN)

Bulimia Nervosa, first described in 1979 by British psychiatrist Gerald Russell as a chronic phase of anorexia nervosa [222], is characterized by overeating and then using compensatory mechanisms, such as self-induced vomiting, laxatives, or prolonged periods of starvation [223]. National studies conducted in the United States estimated the lifetime prevalence of BN ranging between 0.28% and 1.59% [224]. Systematic reviews reported the lifetime prevalence of BN to be 0.81% [199, 225–228]. All eating disorders, including BN, are more prevalent among females and young adults, with a female to male ratio ranging from 3:1 to 8:1 [229, 230].

It seems that microbiota changes modulate appetite regulation in BN patients [231–234]. Unlike AN, a dramatic lack of data is evident for BN. Although BN is also a life-threatening condition, only one study evaluated the role of microbiota in BN, which exclusively focuses on bacterial ClpB protein in patients, without investigating the difference of the gut microbiome species compared with healthy controls [209]. Like AN [235], plasma ClpB level was higher in the patients than in healthy controls. ClpB produced by E. coli is competent in mimicking α-MSH and stimulating an autoimmune response [236, 237]. The difference of BN with AN (hunger rather than satiety) is because of a switch in IgG autoantibody epitope that forms the immunocomplex in BN patients [233, 238].

#### Stress-related functional gastrointestinal disorders (FGIDs)

##### Irritable bowel syndrome (IBS)

Irritable bowel syndrome (IBS) is a chronic functional bowel disorder that is a symptom-based diagnosis as recurrent abdominal pain and changes in stool frequency or form [239, 240]. The global prevalence of IBS is estimated at 11.2% [241]. Sex differences are seen in the prevalence and clinical manifestations of IBS [242]. Irritable bowel syndrome (IBS) is a chronic functional bowel disorder that is a symptom-based diagnosis as recurrent abdominal pain and changes in stool frequency or form [239, 240]. The global prevalence of IBS is estimated at 11.2% [241]. Sex differences are seen in the prevalence and clinical manifestations of IBS [242], but different female to male ratios are reported. Some studies claimed a 2:1 ratio, while others claimed a near 1:1 ratio [243].

The precise mechanism for IBS is not clear. However, evidence show dysregulation of the brain–gut–microbiota interaction may be implicated in the pathophysiology of IBS, and changes in bile acid metabolism have been reported in the patients compared to the healthy population [244–246]. There is evidence of altered metabolism of the gut and dysbiosis in IBS patients, especially the reduction of genera in *Ruminococcaceae* [247]. Also, using probiotics, prebiotics, symbiotics, dietary interventions, and altering the gut microbiota may be an effective way for treating the disease, and certain bacteria may be helpful in IBS treatment [248–250]. The role of the microbiome is probably sex-specific due to shreds of evidence showing that in people using antibiotics, women have a higher risk for IBS. It is also worth noting that this evidence revealed that 12 microbial species differ among IBS patients in a sex-specific manner. After correcting environmental and intrinsic factors that influence the gut microbiome, *Akkermansia muciniphila* was still associated with sex, with higher abundance in females [23]. The role of stress, sex hormones, and the trace aminergic system on the mucosal and the microbiota–brain–gut axis is also an important aspect of IBS [251, 252]. The precise mechanism for IBS is not clear. Stress, sex hormones, and the trace aminergic system on the mucosal and the microbiota–brain–gut axis seem to play an important role in IBS [251, 252]. Dysregulation of the brain–gut–microbiota interaction has been implicated in the pathophysiology of IBS, and changes in bile acid metabolism have been reported in the patients compared to the healthy population [244–246]. There is dysbiosis in IBS patients, especially the reduction of genera in *Ruminococcaceae* [247]. Also, using probiotics, prebiotics, symbiotics, dietary interventions, and altering the gut microbiota may be an effective way for treating the condition, and certain bacteria may be helpful in IBS treatment [248–250]. The role of the microbiome is probably sex-specific, as in people using antibiotics, women have a higher risk for IBS. It is also worth noting that this evidence revealed that 12 microbial species differ among IBS patients in a sex-specific manner. After correcting environmental and intrinsic factors that influence the gut microbiome, Akkermansia muciniphila was still associated with sex, with higher abundance in females [23].

##### Inflammatory bowel disease (IBD)

Inflammatory bowel disease is a chronic inflammation of the gastrointestinal tract, including Crohn’s disease (CD) and ulcerative colitis (UC) [253]. In 2017, more than 6.5 million people were dealing with IBD worldwide [254]. IBD prevalence is sex-dependent, and different traits are seen in various IBD types: UC is 10% more frequent in adult men, whereas CD is 20%–30% more frequent in adult women [255–258]. Interestingly, the prevalence of these two diseases in pediatric patients is the opposite of adults, with the CD being seen more in boys and UC more in girls. This change in the balance of IBDs among male and female patients befalls between 14 and 17 years [259]. IBD can affect physical, psychological, and social aspects of life. As a result, depression, and anxiety are more prevalent in these patients [254].

The exact mechanism of IBD is unclear, but IBD has been associated with microbiota dysbiosis, abnormal inflammatory response, and micronutrients (especially vitamin D) deficiency [260]. Dysbiosis in IBD patients can be described as a reduced population of butyrate-producing species (e.g., *Eggerthella lenta*, *Faecalibacterium prausnitzii*, Clostridium groups IV and XIVa) and Helicobacter spp., and an increased population of Pasturellaceae, Veillonellaceae, Fusobacterium species, and *Ruminococcus gnavus* [260, 261].

Vitamin D plays a role in regulating transcription factors associated with the immune system responses and barrier functions [260], and the Vitamin D receptor is the first human gene known to shape the gut microbiome [262]. Vitamin D deficiency is a common finding in IBD patients, associated with poor outcomes [263]. Murine studies also depict that vitamin D receptor variations can significantly influence the gut microbiota and could have a potential therapeutic role for IBD patients [262, 264]. One meta-analysis in humans revealed that Parabacteroides is the most significant taxon correlated with the VDR gene [262], while a study on Vdr−/− mice revealed enrichment in Clostridium and Bacteroides and depletion in Lactobacillus [265].

Microbiota alterations in IBD patients include a higher number of bacteria on the mucosal surface, a reduction in overall bacterial diversity, and dysbiosis [266]. In ulcerative colitis patients, Gammaproteobacteria, the Enterobacteriaceae (e.g., *Escherichia coli*, *Klebsiella pneumonia*), the Clostridium histolyticum/Clostridium lituseburense group, the Clostridium coccoides/Eubacterium rectale group, the Bacteroides/Prevotella cluster were predominant; while in Crohn’s disease patients, Gammaproteobacteria, Enterobacteriaceae, or Bacteroides/Prevotella was predominant [267–269]. More studies are needed to investigate any sex-specific role of the gut axis in IBD due to contradictory findings: some studies found no separation and difference concerning sex [261, 270], while other studies found sex-specific differences in the microbiome [271] and the association of VDR gene single-nucleotide polymorphism with intestinal pathologies [264].

##### Visceral pain perception

Visceral pain is a heterogeneous spectrum of conditions, ranging from ingestion discomfort to intense renal colic pain [272]. This pain has an enormous effect on life quality affecting sleep, sexual among other functions [273, 274]. Sensing the visceral pain initiates with mechanical stimulation of nociceptors, followed by a nociceptive signal generation that travels within ascending pathways of the spinal cord to thalamic and corticolimbic parts of the brain to be perceived as pain [275].

Gut microbiomes play a pivotal role in the regulation of visceral pain. Different preclinical studies on animals support the role of the gut microbiome in enhanced pain signaling [276, 277]. Germ-free mice studies showed the necessity of gut microbiome for adequate pain sensitivity development since these mice had reduced pain perception following inflammatory stimulation [278]. One new murine study specifically proved that ovariectomy-induced visceral hypersensitivity is dependent on the gut microbiota, and visceral pain is regulated across the estrous cycle in a microbiota-dependent way. Surprisingly this trait was not seen in female germ-free mice, and these mice had similar visceral pain responses to colorectal distension as their conventional controls [279]. So this murine study provides evidence for a significant role of female sex hormones and the gut microbiota in the sensation of visceral pain in females [279], which can also be seen in humans [280].

Some factors such as host genetics, stress, diet, antibiotic consumption, infections, or infancy traumas can assert their roles on visceral pain via disturbing the gut microbiome with an increased population of Lactobacillus (e.g., Lactobacillus paracasei, Lactobacillus reuteri), Bacillus, Bifidobacteria, Clostridium, and Eubacterium rectale [281–284]. Clinical studies also reported altered gastrointestinal microbiota in patients with chronic or recurrent visceral pain (e.g., Inflammatory bowel disease, Irritable bowel syndrome) [266, 285–292]. Other studies on rodents revealed that some bacterial species such as *Lactobacillus paracasei* and *Lactobacillus acidophilus* could induce opioid and cannabinoid receptors in intestinal epithelial cells, which will prevent visceral hypersensitivity and reduce pain perception [281, 293, 294].

### Effect of the gut microbiome and sex differences on neurodegenerative diseases

#### Alzheimer’s disease

Alzheimer's disease (AD) is the most common form of dementia, with an enormous social and economic burden [295, 296]. Alzheimer's disease and other types of dementia are a significant and growing global health challenge, with 40–50 million people currently living with dementia [297]. In 2016, the global prevalence of AD in females was 1·17 times the age-standardized prevalence in males, with more women dying from this condition than men in the same year [297].

The pathological mechanism responsible for AD is complex [298], involving aggregation of the beta-amyloid peptide and hyperphosphorylated tau protein (also called neurofibrillary tangles or NFTs) and loss of neuronal connections in the brain, which results in loss of brain function [299–302]. Autopsy studies have shown a more significant global AD pathology burden in women than in men because of higher loads of NFTs [303, 304].

Gut microbiome alteration is somehow involved in Alzheimer's disease, similar to other related neurodegenerative diseases (e.g., Parkinson's disease) [305]. For instance, Vogt et al. reported that trimethylamine *N*-oxide, a microbiota-derived metabolite, was elevated in Alzheimer's patients [306].

Zhuang et al. and Vogt et al. independently looked at the gut microbiome of patients with Alzheimer's disease, with a lower abundance of Firmicutes and Bifidobacterium and a higher abundance of Bacteroidetes, Proteobacteria, and Actinobacteria than healthy individuals [307].

One murine study specifically looked into microbiome alterations sex-specifically, reporting a higher abundance of Prevotella and Ruminococcus in female mice and a lower abundance of Sutterella than male mice [308].

#### Parkinson’s disease

Parkinson’s disease (PD) is a neurodegenerative disease characterized by bradykinesia, rigidity, resting tremor and postural instability [309]. Based on the global burden of disease 2016 estimations, 6.1 million (95% UI 5–7.3) people suffered from PD worldwide. Of those, 2.9 million (47.5%) were women, and 3.2 million (52.5%) were men [310]. The primary pathogenesis of PD is suggested as the loss of dopaminergic neurons in the basal ganglia (especially substantia nigra) and spreading Lewy bodies through various brain zones [311].

Several studies have provided evidence on gut microbiota alterations in individuals with PD compared to healthy controls [312–314], describing reduced abundances of *Dorea, Bacteroides, Prevotella, Faecalibacterium, Bacteroides massiliensis, Stoquefichus massiliensis, Bacteroides coprocola, Blautia glucerasea, Dorea longicatena, Bacteroides dorei, Bacteroides plebeus, Prevotella copri, Coprococcus eutactus,* and *Ruminococcus callidus* [313]. Also, increased abundances of *Christensenella, Catabacter, Lactobacillus, Oscillospira, Bifidobacterium, Christensenella minuta, Catabacter hongkongensis, Lactobacillus mucosae, Ruminococcus bromii,* and *Papillibacter cinnamivorans* are reported [313].

Interestingly, Braak’s seminal theory stated that an unknown neurotropic factor goes through the gut and causes progressive increments in Lewy body pathology. Moreover, Lewy pathogens spread in the brain via the vagus nerve [315]. Several human and experimental studies elaborated on this theory and showed evidence that gut might indeed instigate PD pathogenesis [316].

Sex hormones, primarily estrogens, are thought to play an essential role in protecting healthy cells and neurons against oxidative mechanisms, also maintaining the dopaminergic system functions [317, 318]. Notably, estrogen is assumed to be an origin for existing sex differences in PD because of its protective roles [319]. In an experimental study by Siani et al. [320], female mice revealed a higher dopaminergic loss in the substantia nigra due to ovariectomy compared to controls. Interestingly, exogenous estrogen led to a preserved dopaminergic loss, which could be considered as a potential therapeutic agent in PD patients. Considering the roles of the gut–brain axis in the mechanism of action of levodopa (primary PD treatment) [321] and the roles of estrogen, bring us this idea that sex-specific therapeutic agents affecting the gut–brain axis may be effective. Apart from several studies focusing on microbiome and PD, and sex and PD interactions, no studies have not been performed to exhibit sex-specific roles of microbiota–gut–brain axis in the occurrence and progression of PD.

#### Multiple sclerosis

Multiple sclerosis (MS) is an autoimmune proinflammatory CNS-demyelinating disease [322] involving genetic and environmental factors in its pathology [323, 324]. The Global Burden of Disease Study reported that there were 2,221,188 prevalent cases of multiple sclerosis in 2016 globally (95% uncertainty interval [UI] 2 033 866–2 436 858) [325], which indicates that every 5 min, someone, somewhere in the world is diagnosed with MS [326]. The global prevalence of MS in adults varies considerably by sex, with the sex ratio of 2:1 in favor of women in the sixth decade of life [325].

Recent evidence implies that gut microbiota is one of the critical environmental factors in MS etiology [327], and different studies are done on the microbiome profile of MS patients. Zhang et al. find that GI problems (e.g., constipation, bloating, fecal incontinence) and small intestinal bacterial overgrowth are more common in MS patients compared with sex and age-matched controls without MS [328]. Chen et al. reported an increased abundance of Pseudomonas, Mycoplana, Haemophilus, Blautia, and Dorea and a decreased abundance of Parabacteroides, Adlercreutzia, and Prevotella genera in MS patients compared with healthy controls [327]. Tremlett et al. study on pediatric patients revealed significant enrichment in the relative abundance of the Desulfovibrionaceae (Bilophila, Desulfovibrio, and Christensenellaceae) and depletion in Lachnospiraceae and Ruminococcaceae; however, overall gut bacterial beta diversity was not significantly related to the disease status [329]. Another study on progressive MS and relapsing–remitting MS found increased Clostridium bolteae, Ruthenibacterium lactatiformans, Akkermansia, and decreased Blautia wexlerae Dorea formicigenerans, and Erysipelotrichaceae CCMM [330].

Regarding the sex differences, one murine study on the effect of Alcohol on autoimmune encephalomyelitis (the most commonly used experimental model for the human inflammatory demyelinating disease) noted that moderate alcohol consumption significantly lessens clinical EAE Severity in a Sex-Specific Pattern: alcohol-fed males underwent more significant disease remission, male-specific decrease in microglial density in the cervical and thoracic spinal cord in late-stage disease, and sex-specific alterations in essential microbiota known for their immune regulatory roles (including more increment of Turicibacter, Akkermansia, Prevotella, and Clostridium in females compared to males) [331]. In humans, Chen et al. find that the microbes involved in the phytoestrogen metabolic pathway (Prevotella, Parabacteroides, and Adlercreutzia) are interestingly increased in female patients [327]. However, more studies are needed to mark the possible role of sex in the microbiome changes of MS patients.

## Probiotics, prebiotics, and dietary interventions based on sex

As diet plays an essential role in forming the gut microbiota composition, several clinical trials and cohorts are performed to reveal how diet can affect the brain and behavior via altering gut microbiota pattern [64]. Probiotics including Lactobacillus and Bifidobacterium species have been evaluated as therapeutic agents in various mental and neurodegenerative disorders [332]. Preclinical studies of the administration of probiotics in ASD children revealed promising findings, but a meta-analysis of human studies showed that these agents might not help relieve GI and behavioral symptoms [333]. In addition, another meta-analysis reviewing the efficacy of probiotics in patients with schizophrenia failed to show any promising outcomes. However, probiotics help improve the metabolic effects of antipsychotic medications [334]. Prebiotics and probiotics have been administered for depression and anxiety. Aggregated data of 34 randomized controlled trials supported the potential antidepressant and anxiolytic effects of probiotics [335].

Dementia and AD is another neuropsychiatric disorder in which the effects of probiotics have been assessed. However, based on the results of a meta-analysis, data were not adequate to firmly conclude that they have positive effects [336, 337]. Additionally, daily utilization of strains of Lactobacillus and during the exams in students decreased diarrhea and increased sleep quality in men and relieved stress-related somatic symptoms in women [338].

Regarding sex differences, a study on a large sample of wild and laboratory fishes, laboratory mice, and humans evaluated the interactive mechanism of action of sex and diet on the gut microbiota composition. This elegant study reported that some species which were responsive to sex hormones also responded to foods variations [21]; for instance, mice revealed that diet might alter the gut microbiota in males and females dissimilarly. Considering the two natural fish populations, correlations between the individual differences and diet disparities among subjects were observed [21]. Consistently, a low-fat diet caused an increased abundance of *Desulfovibrio, Roseburia,* and *Holdemania* in males compared to females [339]. Moreover, in line with this idea that dietary-based interventions might alter sex hormones levels and regulations, serum levels of estradiol and progesterone were contrarily associated with the utilization of dietary fibers in women [340]. In addition, male mice supplemented with Lactobacillus showed higher testosterone levels and matured and larger testicles compared to the untreated [341].

## Socioeconomic status (SES) and its effects on neuropsychiatric disorders

Low socioeconomic status (SES) affects different areas of social life. Investigating the role of SES on psychiatric disorders is not something new, and some old studies exist on this matter [342, 343]. SES indexes and various socioeconomic status markers are defined for these studies, such as position in the labor market, occupation status, education status, household income, poverty, material wealth, or family affluence [344].

Various studies analyzed the role of sex on this theme. Multiple studies did not find any evidence for sex differences between SES and mental health disorders [345–347]. Leve et al. [348] found evidence that girls from low-SES families are affected more than boys, while Due et al. [349] and Lipman et al. [350] had stated the opposite. One meta-analysis studying the effect of socioeconomic status (SES) on the mental health of children and adolescents aged four to 18 years found that socioeconomically underprivileged individuals were two to three times more likely to develop mental health problems [351]. These contradictory results show the need for more large-scale studies in this area.

A recent study revealed that the children of a parent with a mental disorder were at more risk of presenting mental disorders than those without these problems. Interestingly, the mother's SES and mental disorders showed a higher association with the child's mental disorders than the father's [352].

The mechanism by which SES affects mental health is not entirely understood, but it is suspected that some genes and hormones are involved. Epigenetic modification, particularly methylation of gene regulatory regions, shapes human brain function associated with risk for mental illness [353]. As for the role of hormones, Zhu et al. found low-SES children showed an increase in pre-bedtime basal cortisol but a decrease in the cortisol awakening response [354].

## Future directions and conclusion

Considering the effects and specificity of the microbiome in human diseases, new therapeutic approaches targeting gut microbiota have been developed to improve human health. Within the precision medicine framework, the microbiome seems an essential element. For personalized drug therapies, the microbiome's effect on the metabolism, availability, efficacy, and toxicity should be taken into account.

**Fig. 2:**
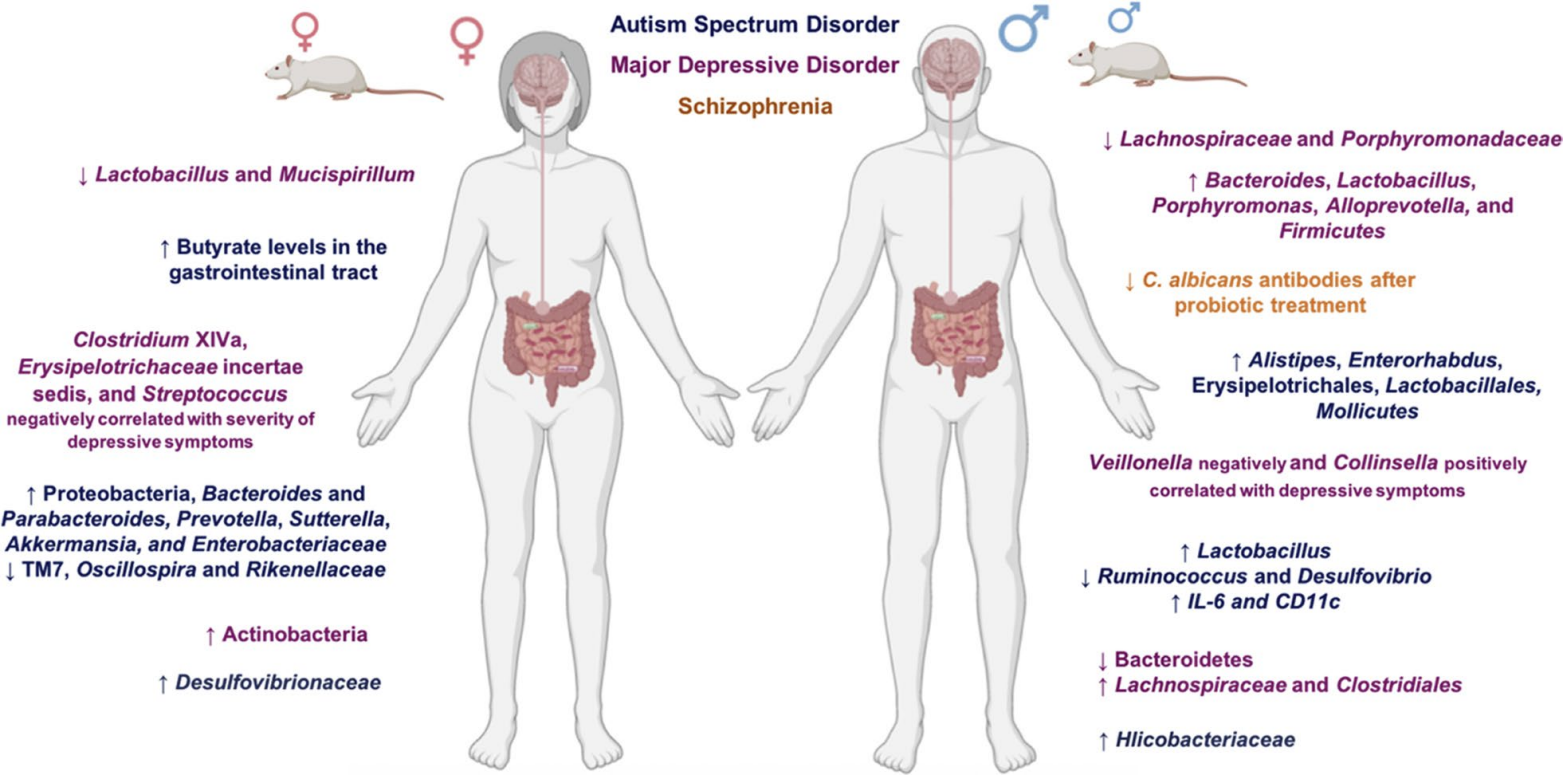
Gut-microbiota pattern changes associated to sex and neuropsychiatric disorders. (Letters in blue colors are associated with autism spectrum disorder, purple with major depressive disorder, and orange with schizophrenia.) (Reprinted from Manosso et al. [355] with copyright permission from Elsevier under License number 5158380038251. https://www.sciencedirect.com/science/article/abs/pii/S0361923021000988.)

Although many aspects of the effects of sex differences in the brain and gut microbiota composition have been revealed, these interactions and their relationship to the incidence of neuropsychiatric disorders remain unclear. Studies are needed to explore the associations between sex differences and microbiota in conditions including PD, MS, ADHD, PTSD, GAD, bulimia, and anorexia nervosa. In addition, most studies focus on male features; thus, experiments revealing female characteristics are more needed in this regard. To conclude, sex differences and gut microbiota are bidirectionally affecting each other, and these communications may lead to the occurrence and progression of specific neuropsychiatric disorders. Furthermore, sex can influence gut microbiota composition and diversity via multiple pathways and mechanisms, resulting in different incidence rates of neurologic and psychiatric disorders. Figure 2 summarizes sex-related differences in gut microbiota and their possible relations to various neuropsychiatric disorders.

## Data Availability

Not applicable.

## Abbreviations

AD: Alzheimer’s disease
ADHD: Attention deficit hyperactivity disorder
AN: Anorexia nervosa
ASD: Autism spectrum disorder
BN: Bulimia nervosa
CD: Crohn’s disease
ClpB: Caseinolytic proteinase B
FGID: Functional gastrointestinal disorders
FMT: Fecal microbiota transplantation
GAD: Generalized anxiety disorder
GBA: Gut–brain axis
IBD: Inflammatory bowel disorder
MDD: Major depressive disorder
MS: Multiple sclerosis
NFT: Neurofibrillary tangles
PD: Parkinson’s disease
PTSD: Post-traumatic stress disorder
SCFA: Short-chain fatty acids
SPF: Specific pathogen-free
UC: Ulcerative colitis
WHO: World Health Organization
